# Erythropoietin mitigated thioacetamide-induced renal injury via JAK2/STAT5 and AMPK pathway

**DOI:** 10.1038/s41598-023-42210-1

**Published:** 2023-09-11

**Authors:** Marawan A. Elbaset, Bassim M. S. A. Mohamed, Shaimaa A. Gad, Sherif M. Afifi, Tuba Esatbeyoglu, Sahar S. Abdelrahman, Hany M. Fayed

**Affiliations:** 1 Pharmacology Department, Medical Research and Clinical Studies Institute, National Research Centre, 33 El-Bohouth St., Dokki, P.O. 12622, Cairo, Egypt; 2 Pharmacognosy Department, Faculty of Pharmacy, University of Sadat City, Sadat City, 32897 Egypt; 3 Department of Food Development and Food Quality, Institute of Food Science and Human Nutrition, Gottfried Wilhelm Leibniz University Hannover, Am Kleinen Felde 30, 30167 Hannover, Germany; 4 Department of Pathology, Faculty of Veterinary Medicine, Cairo University, Giza, Egypt

**Keywords:** Kidney, Kidney

## Abstract

The kidney flushes out toxic substances and metabolic waste products, and homeostasis is maintained owing to the kidney efforts. Unfortunately, kidney disease is one of the illnesses with a poor prognosis and a high death rate. The current investigation was set out to assess erythropoietin (EPO) potential therapeutic benefits against thioacetamide (TAA)-induced kidney injury in rats. EPO treatment improved kidney functions, ameliorated serum urea, creatinine, and malondialdehyde, increased renal levels of reduced glutathione, and slowed the rise of JAK2, STAT5, AMPK, and their phosphorylated forms induced by TAA. EPO treatment also greatly suppressed JAK2, Phosphatidylinositol 3-kinases, and The Protein Kinase R-like ER Kinase gene expressions and mitigated the histopathological alterations brought on by TAA toxicity. EPO antioxidant and anti-inflammatory properties protected TAA-damaged kidneys. EPO regulates AMPK, JAK2/STAT5, and pro-inflammatory mediator synthesis.

## Introduction

The kidney plays a crucial role in the elimination of harmful chemicals and metabolites, as well as the preservation of homeostasis^1^. One of the diseases with a high mortality rate and a life-threatening prognosis is kidney disease^2^. Failure of the kidneys is regarded as a serious health issue on a global scale^3^. High morbidity and mortality are associated with acute renal injury (AKI), a serious illness marked by a rapid renal function reduction^4^. In addition, AKI has a high chance of developing chronic kidney disease and end-stage renal disease, characterized by tubulointerstitial fibrosis^5^. Nephrotoxicity is regarded as one of the most common kidney problems and occurs when the body is exposed to a drug or toxin^6^.

Thioacetamide (TAA) is a potent hepatotoxin initially used as a fungicide^7^. TAA is the most powerful nephrotoxic agent due to its cumulative harm when delivered intermittently^8^. The mixed-function oxidase system metabolizes TAA to hazardous metabolites, which are subsequently disseminated throughout several tissues^9,10^. TAA toxicity is theorized to originate from thioacetamide sulfoxide (TAAS) and its conversion to thioacetamide dioxide (TAAD), both of which are poisonous to the kidneys and liver^11^.

One of the primary signaling pathways thought to regulate cytokine production involves the Janus kinase (JAK) family and signal transducers and activators of transcription (STATs), which has been linked to renal illness^12^. JAK1, 2, and 3 are members of the JAK family, whereas STAT1, 2, 3, 4, 5, and 6 are members of the STAT family. Both families have been confirmed in mammals. Animals AKI or ischemia–reperfusion (I/R) damage models have demonstrated the involvement of JAK 1 and 2 as well as STAT 3, 5, and 6 in the development of AKI^13^. It is generally recognized that many cytokines, including TNF-α and IL-6, have a role in the immune response via the JAK/STAT pathway^14^. The JAK/STAT system is a crucial member of intracellular signal transduction pathways involved in cellular death, inflammatory responses, and oxidative stress responses^15^. The JAK/STAT pathway is also implicated in the edaravone action that treats kidney disorders such as obstructive nephropathy, diabetic nephropathy, and different types of glomerulonephritis^16^. As a result, blocking the JAK/STAT pathway is an effective treatment strategy for renal injury.

Noteworthy, Phosphatidylinositol 3-kinases (PI3k) are a family of enzymes involved in cellular functions such as cell growth, proliferation, differentiation, motility, survival, and intracellular trafficking^17^. Class I PI3Ks activate protein kinase B (PKB, aka Akt) in the PI3K/AKT/mTOR pathway, which is critical for a wide variety of cellular processes^18^. It is widely established that pathogenic activation of the PI3K/Akt/mTOR network causes uncontrolled cell growth. Multiple disorders, including cancer, have had pharmacological targets set on this route. The mTOR cascade increases cyst formation in polycystic kidney disease (PKD) by increasing tubule epithelial cell proliferation, size, and metabolism^19^.

Additionally, The Protein Kinase R-like ER Kinase (PERK) is an important protein located in the ER that regulates signal transduction during ER stress. Liver fibrosis, atherosclerosis, ischaemic heart disease, and myocardial damage are all caused by the ER stress response that is triggered by a number of different injuries^20^. Given that the ER stress response is a crucial cause of many liver diseases, it is vital to discover medications that efficiently inhibit it.

The kidney produces defensive reactions in response to injury, limits cell injury, and boosts its repair, one of which is linked to the erythropoietin hormone (EPO)^21^. EPO is a hematopoietic hormone produced in response to hypoxia and is a crucial protein in erythrocyte formation. In response to hypoxia, it primarily increases neovascularization and angiogenesis, improving blood flow and primarily manifesting in the kidney. EPO physiological effects are mediated by its ability to bind to EPO receptors (EPORs), which can be found in various tissues and cells, such as mesangial, glomerular, and tubular epithelial cells^22^. EPO has several significant impacts, including a decrease in free radicals and pro-inflammatory cytokines. Recombinant human EPO (rHuEPO) reduces lipid peroxidation linked to oxidative stress, the infiltration of inflammatory cells, and the apoptotic process. In addition, it is revealed that EPO may protect against ischemia/reperfusion-induced damage to the liver, heart, and lungs^23^. In several models of physiological insults, EPO was shown to be protective, and the induction of protection was through JAK2/STAT5 pathway^24^.

Therefore, this study was designed to evaluate the protective effect of EPO administration against renal injury induced via TAA. In addition, the involvement of JAK2/STAT5 signaling in mediating the protective effect of EPO was also evaluated.

## Materials and methods

### Animals

Twenty-four albino male rats weighing 185–205 g were purchased from “Animal House at the National Research Centre (NRC, Cairo, Egypt). Rats were maintained under standard conditions of temperature (25 °C) with a 12 h (light)–12 h (dark) cycle. The animals were treated according to national and international ethics guidelines. The ethics committee has approved all procedures and experiments of the NRC (Cairo, Egypt)”^25^.

The sample size was calculated using G-Power software version 3.1.9.4 (Fraz faul, Germany). The study has 4 independent groups. Prior data indicated a difference in serum creatinine (sCr) in the kidney-induced injury group is approximately 4-fold compared to a normal control group, according to previous studies in our lab. Twenty rats were assigned to each study group to achieve an effect size (f) of 1.08 and a study power of 95% (1- β error probe). A 20% increment in the sample was added to avoid any expected death. Therefore 24 rats were utilized to reject the null hypothesis that the effect of the drug-treated group and normal control groups are equal. A continuity-corrected squared Fishers exact test will be used to evaluate this null hypothesis with a probability of type I error (α error = 0.05), power = 95%.

### Experimental design

**Figure 1:**
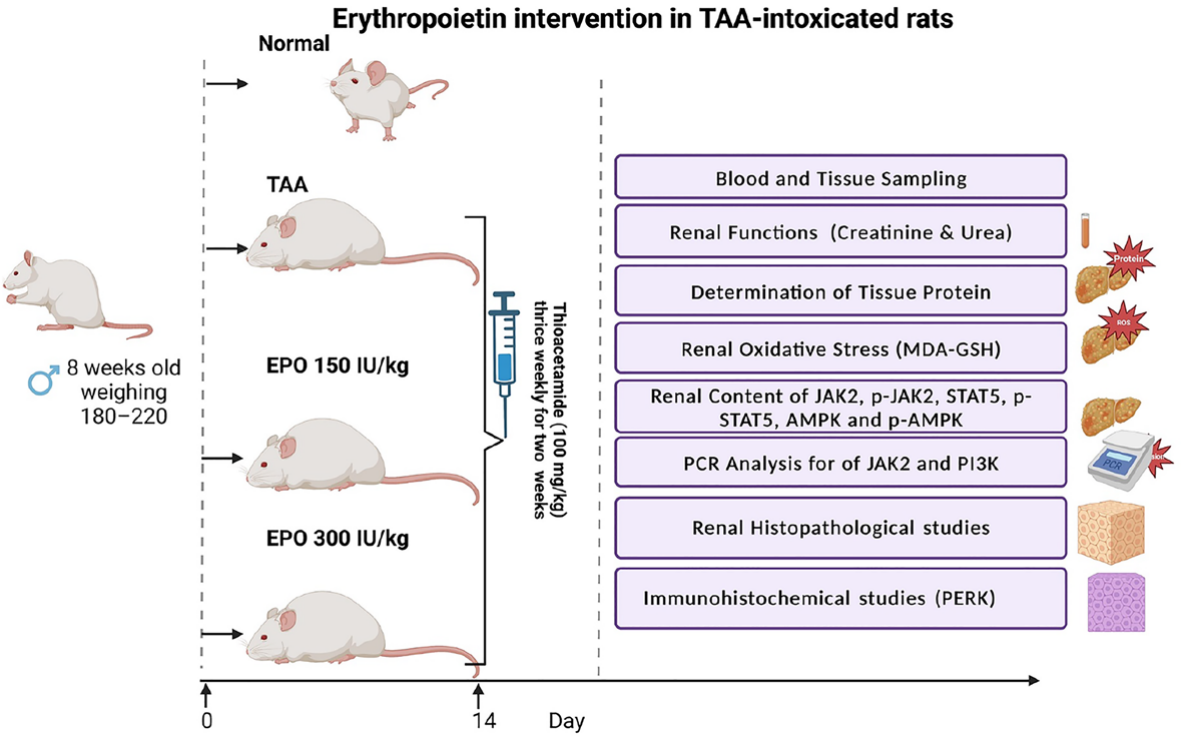
Experimental design of EPO intervention in TAA-intoxicated rats.

Injection of TAA intraperitoneally (IP) was used to induce renal damage, as previously described^26^. The rats were randomly spread into four sets (6 rats/set). Rats in group 1 (negative control group) were given an intraperitoneal (IP) injection of saline three times a week for two consecutive weeks. Rats in group 2 (TAA group) received an intraperitoneal (IP) injection of TAA (100 mg/kg) three times a week for two consecutive weeks to cause renal injury^26^. Rats in groups 3 and 4 were given intraperitoneal injections of EPO (150 and 300 IU/kg)^27^ every day for two weeks following the TAA injection, as seen in Fig. 1.

### Preparation of blood samples

“Rats were fasted overnight at the end of the experiment, and then blood samples were collected, and the serum was obtained by centrifugation at 3000 rpm at 4°C for 5 min and stored at − 80°C, to be used later on for the measurements of blood urea and creatinine as kidney function tests” as described by^28^.

### Renal tissue collection

In a similar manner, “the renal tissue was dissected, washed with ice-cold saline, aliquoted, and divided into three parts. The first part was instantly frozen in liquid nitrogen and stored at − 80°C for mRNA extraction to estimate the gene expression of JAK2 and PI3K.

The second part was homogenized (100 mg of tissue: 1 mL of iced 0.5% potassium chloride) followed by 1-min sonication and 10 min centrifugation at 3000 rpm at 4°C, and finally, the supernatant was separated and preserved at − 80°C for the measurement of renal GSH and MDA using colorimetric “Bio-diagnostic® kits, Cairo, Egypt; Cat# MD 25 29, GR 25 11 and SD 25 21)” according to the method of Metwaly^28^. At the same time, the measurements of JAK2, STAT5, AMPK, and p-AMPK were assessed using enzyme-linked immunosorbent assay (ELISA).

The last part was fixed in 10% buffered formalin for further histopathological and immune-histochemical investigations^29,30^.

### Renal function tests

Serum urea was used to evaluate renal function (Catalog# K375-100, BioVision, Milpitas Boulevard, Milpitas, USA), as well as serum creatinine (Catalog# E4370-100, BioVision, Milpitas Boulevard, Milpitas, USA) levels, were measured by colorimetrically using a commercial kit.

### Assessment of Lipid peroxidation in renal tissue

The measurement of MDA level (Catalog# K739-100, BioVision, Milpitas Boulevard, Milpitas, USA) and GSH content (Catalog# K464-100, BioVision, Milpitas Boulevard, Milpitas, USA) was performed colorimetrically according to the manufacturer instructions.

### Renal JAK2 and PI3K gene expression in renal tissue

Thermo Fisher Scientific (Massachusetts, USA) supplied the SuperScript IV One-Step RT-PCR kit (Cat# 12594100) for reverse transcription and PCR of the isolated RNA. A thermal profile was conducted using a 96-well plate StepOne equipment (Applied Biosystem, California, USA) as follows: Reverse transcription takes 10 min at 45 °C, RT inactivation takes 2 min at 98 °C, and the initial denaturation phase requires 40 cycles of 10 s at 98 °C, 10 s at 55 °C, and 30 s at 72 °C for the amplification step. According to Nada et al., normalization for variation in the expression of target genes JAK2 and PI3K was performed by referring to the mean critical threshold (CT) expression values of the GAPDH housekeeping gene by the ΔΔCt method and the relative quantitation (RQ) of each target genes was quantified according to the calculation of 2^−∆∆Ct^ method”. Table 1 presents the oligonucleotide sequences for the forward and reverse primers^31^.

**Table 1:** List of oligonucleotide primers used in qPCR.

Gene		Sequence (5′-3′)	
*JAK2*	F	TCCACCCAATCATGTCTTCCA	NM_031514.1
	R	ATGGTGTGCATCCGCAGTTA	
*PI3K*	F	TGGCAGTTCAAAGCGAAACC	XM_032898971.1
	R	TCATGGTGGGGCAAATCCTC	
*GAPDH*		CAATCCTGGGCGGTACAACT	XM_039107008.1
		GATGGTGATGGGTTTCCCGT	

### Renal JAK2, STAT5, AMPK, and P-AMPK levels

To quantify protein quantities, we utilized commonly available ELISA kits of JAK2 and p-JAK2 (Catalog# abx050224 and abx333115 Abbexa LLC, Houston, TX USA) as well as STAT5 and p-STAT5 (Catalog# NBP2-80282, Novus Biologicals, LLC, Denver, USA, and Catalog #: and PEL-Stat5-Y-1 RayBiotech, Georgia, USA), AMPK and p-AMPK (Catalog# MBS1602983, MBS164089, MyBioSource, San Diego, USA) in renal tissue homogenate. The manufacturer instructions were followed for each ELISA kit.

### Histopathological examinations

In 10% buffered formalin, renal tissues were fixed for 24 h. Then, it is dehydrated in different grades of alcohol, cleared in xylene, and embedded in paraffin wax. Using hematoxylin and eosin (H&E) stain, the paraffin sections (4 μm) were stained. To avoid bias, the sections were examined by a pathologist who was blinded in the coding of sections using a light microscope.

### Immune-histochemical examination of PERK expression

The other paraffin section from each group was used for immunohistochemical detection of the expression of PERK in various experimental groups using avidin–biotin peroxidase according to method described by^32^. To detect antigen–antibody complexes, kidney slices were treated with monoclonal antibodies for PERK (Abcam, Cambridge, MA, USA) at a dilution of 1:200 (Vactastain ABC peroxidase kit, Vector Laboratories). Chromagen diaminobenzidine tetrahydrochloride was used to visualize each marker expression (DAB, Sigma-Aldrich, St. Louis, MO, USA). The positive brown area of each marker expression was measured using Image J, 1.46a analysis software (NIH, USA), and seven high-power microscopic fields.

### Statistical analysis

The statistics were performed by presenting the Data “as mean ± S.E, and analyzed by one-way ANOVA followed by the Tukey–Kramer Post hoc test in case of normality, and were analyzed using the Kruskal–Wallis test followed by Dunn’s multiple comparisons test in case of non-normality (GraphPad Software, version 9, USA). The significance limit was set to *p* < 0.05”^33^.

### Ethics approval and consent to participate

The National Research Centre, Medical Research ethics committee has approved all procedures and experiments with permit no. “1041112022”. The study is reported in accordance with ARRIVE guidelines.

## Results

### Impact of EPO on renal parameters indicators of TAA-intoxicated rats

**Figure 2:**
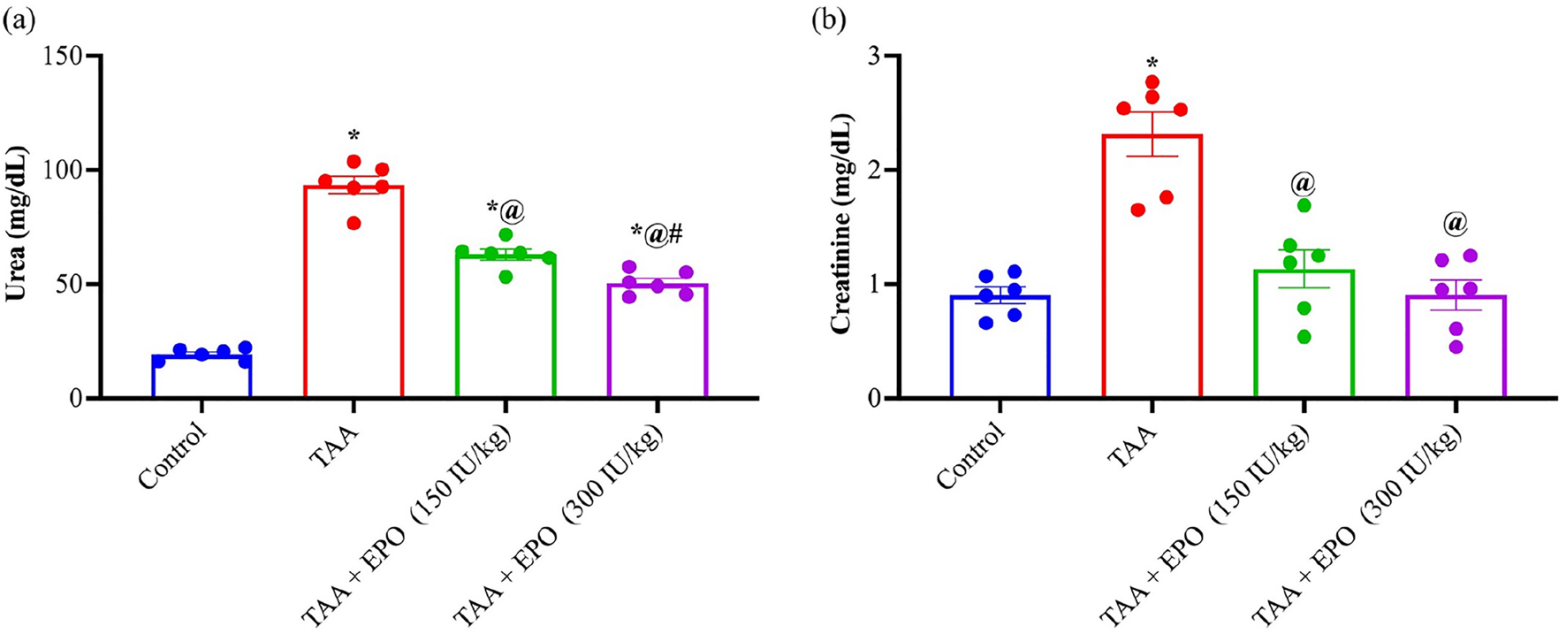
Effect of EPO (150 and 300 IU/kg) on serum renal function tests of TAA-intoxicated rats (**a**) urea and (**b**) creatinine. Each bar represents the mean ± SE of 6 rats. * vs normal control group, @ vs TAA group, # vs EPO (150 mg/kg) at *p* < 0.05.EPO: Erythropoietin; TAA: thioacetamide.

Creatinine and urea levels in the blood were dramatically elevated by 4.8 and 2.6-fold compared to the normal group after TAA was administered, as seen in Fig. 2. Conversely, both EPO treatment groups substantially lowered blood creatinine and urea levels after EPO delivery.

### Effect of EPO on oxidative stress markers in renal tissue of TAA-intoxicated rats

**Figure 3:**
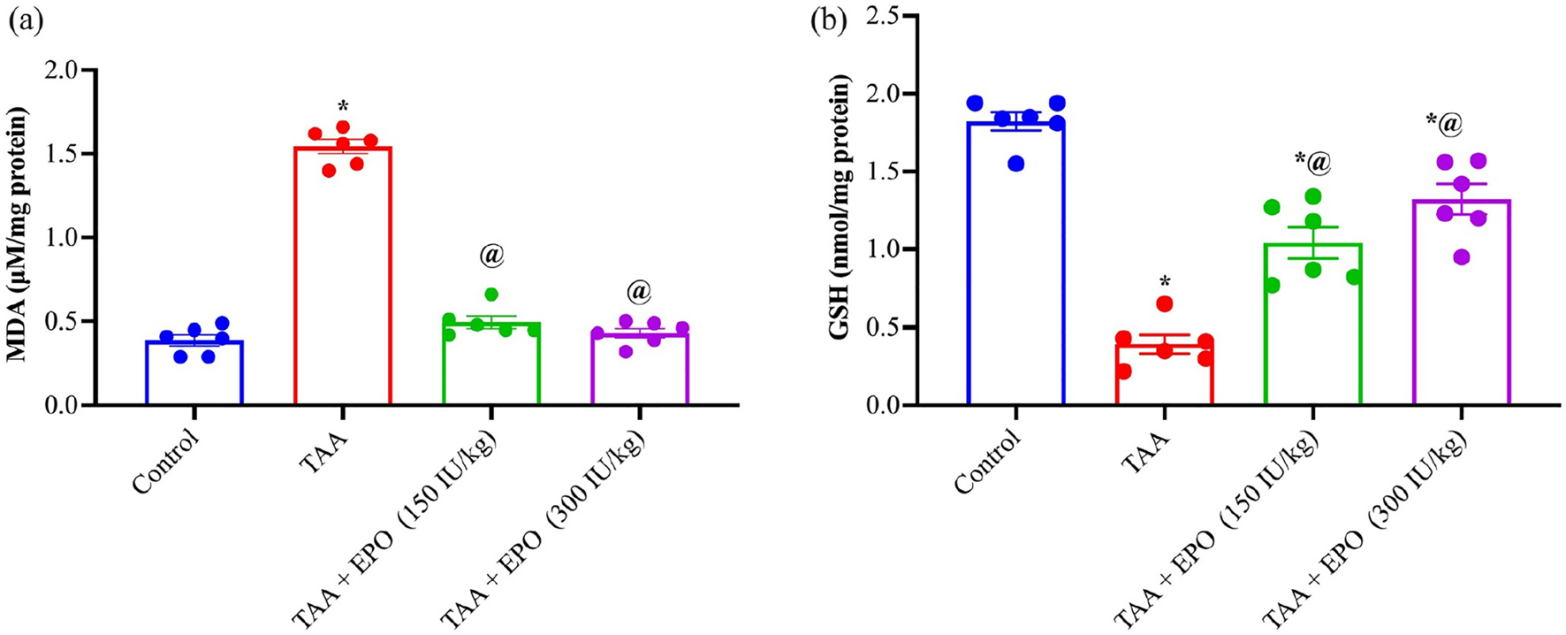
Effect of EPO (150 and 300 IU/kg) on oxidative stress markers in renal tissue of TAA-intoxicated rats (**a**) MDA (µM/mg protein) (**b**) GSH (nmol/mg protein). Each bar represents the mean ± SE of 6 rats. ^*^ vs normal control group, ^@^ vs TAA group, ^#^ vs EPO (150 mg/kg) at *p* < 0.05. EPO: Erythropoietin; TAA: thioacetamide.

Compared to the control group, those receiving TAA had marked higher levels of MDA by 4-fold in their kidneys and reduced levels of GSH by 78% in their kidneys. Renal MDA content was substantially reduced by EPO treatment in both treated groups by 68 and 72%, and GSH was also replenished by 2.6 and 3.3-fold compared to the TAA group. As demonstrated in Fig. 3, EPO led to enhanced antioxidant activity.

### Effect of EPO on renal content JAK2/P- JAK2, STAT5/P-STAT5, and AMPK/P-AMPK in renal tissue of TAA-intoxicated rats

**Figure 4:**
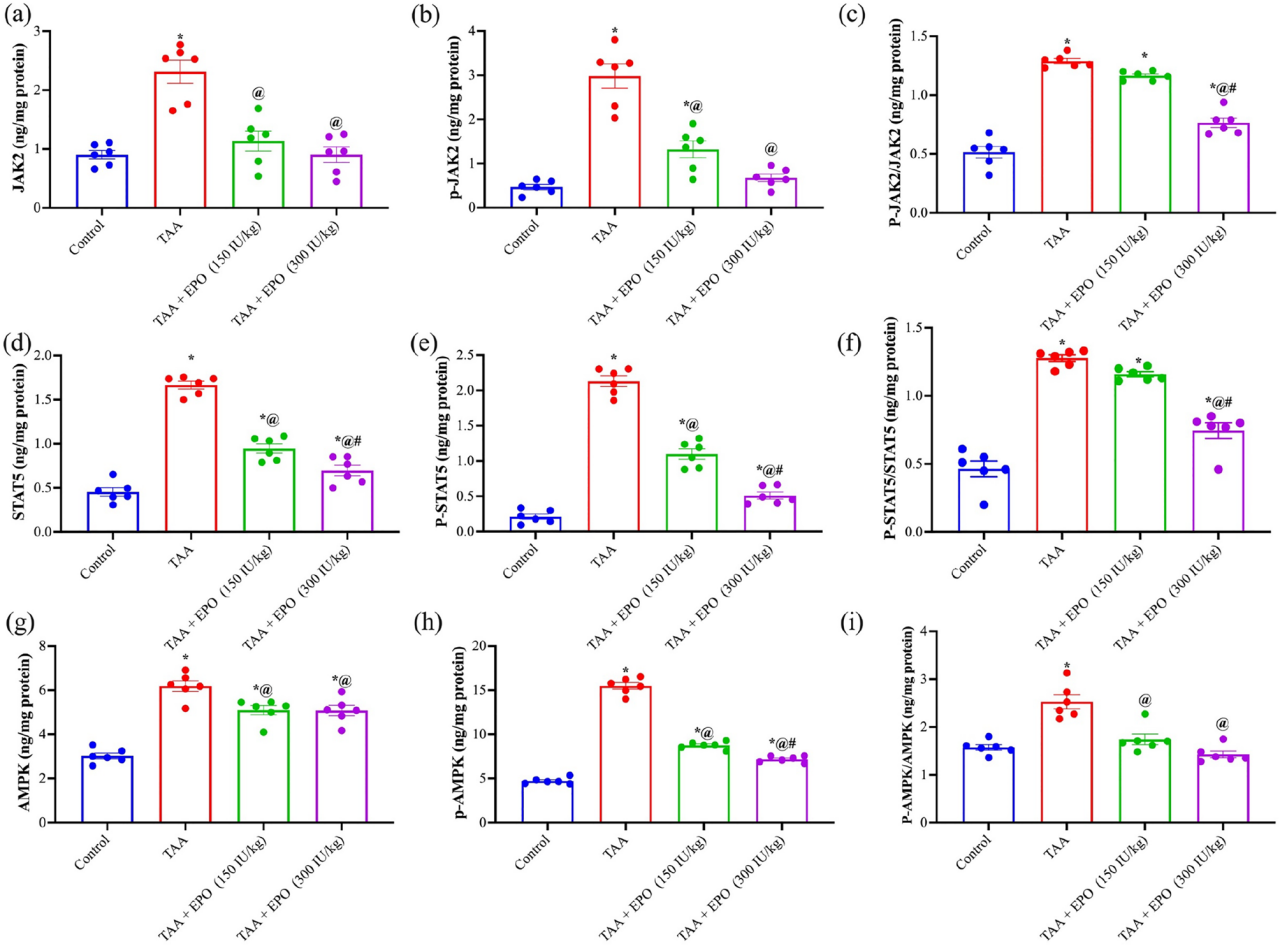
Effect of EPO on renal content of (**a**) JAK2 (**b**) p- JAK2, (**c**) p- JAK2/JAK2, (**d**) STAT5, (**e**) p-STAT5, (**f**) p-STAT5/STAT5, (**g**) AMPK, (**h**) p-AMPK and (**i**) p-AMPK/AMPK in renal tissue of TAA-intoxicated rats. Each bar represents the mean ± SE of 6 rats. * vs normal control group, @ vs TAA group, # vs EPO (150 mg/kg) at *p* < 0.05.EPO: Erythropoietin; TAA: thioacetamide; GSH: reduced Glutathione; MDA: Malondialdehyde.

Renal damage caused by TAA led to a dramatic rise in the protein levels of JAK2/p- JAK2, STAT5/p-STAT5, and AMPK/p-AMPK by 2.5, 2.75, and 1.6-fold related to the control group. At the same time, only EPO (300 IU/kg) administration drastically reduced JAK2/p- JAK2, STAT5/p-STAT5, and AMPK/p-AMPK by 40, 42 and 43% compared to TAA, as shown in Fig. 4.

### Effect of EPO on renal JAK2 and PI3K gene expression of TAA-intoxicated rats

**Figure 5:**
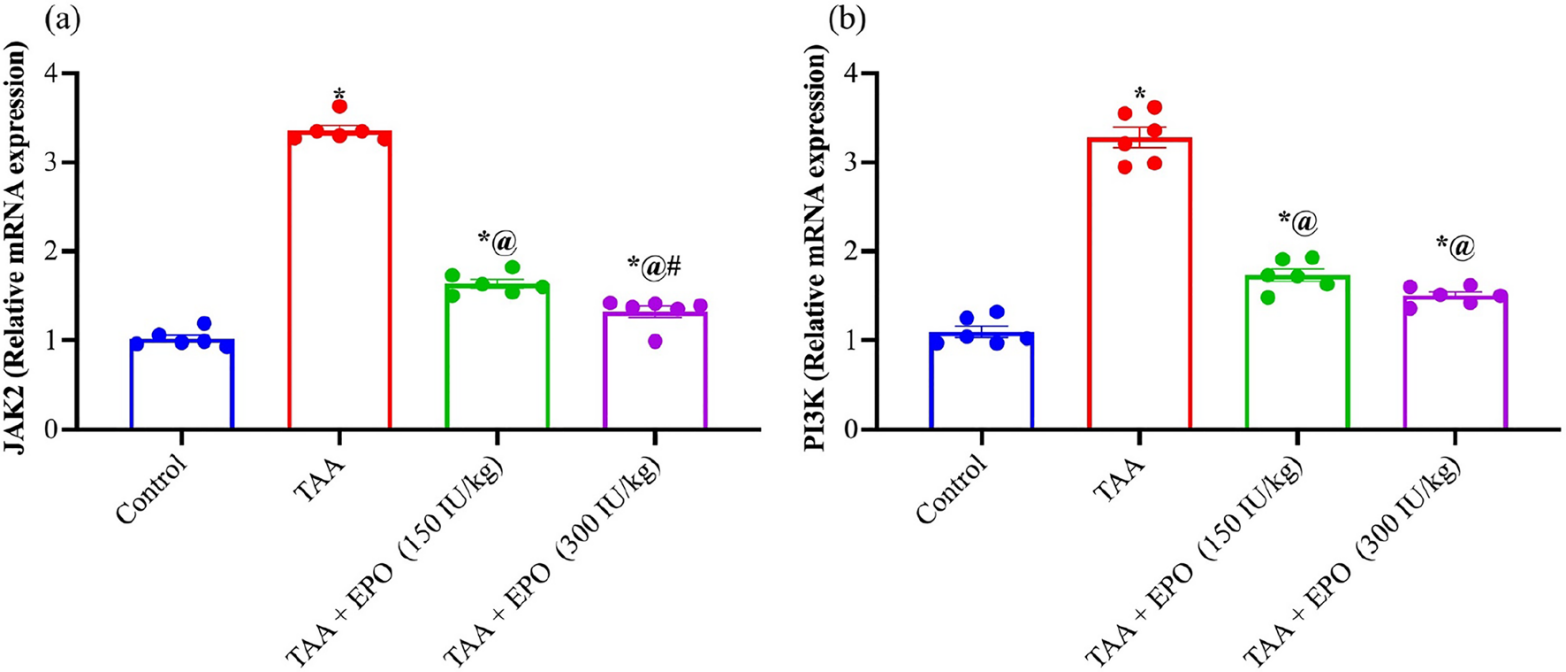
Effect of EPO on renal gene expression of (**a**) JAK2 and (**b**) PI3K of TAA-intoxicated rats. Each bar represents the mean ± SE of 6 rats. * vs normal control group, @ vs TAA group, # vs EPO (150 mg/kg) at *p* < 0.05.EPO: Erythropoietin; TAA: thioacetamide.

Gene expression levels for JAK2 and PI3K were upregulated by 3.3 and 3.2 following TAA treatment compared to the control group. Rats treated with EPO (150 or 300 IU/kg) showed lower levels of JAK2 by 51 and 60% as well as PI3K by 47 and 54% gene expression than the TAA-injured rats, respectively, as shown in Fig. 5.

### Histopathology findings

**Figure 6:**
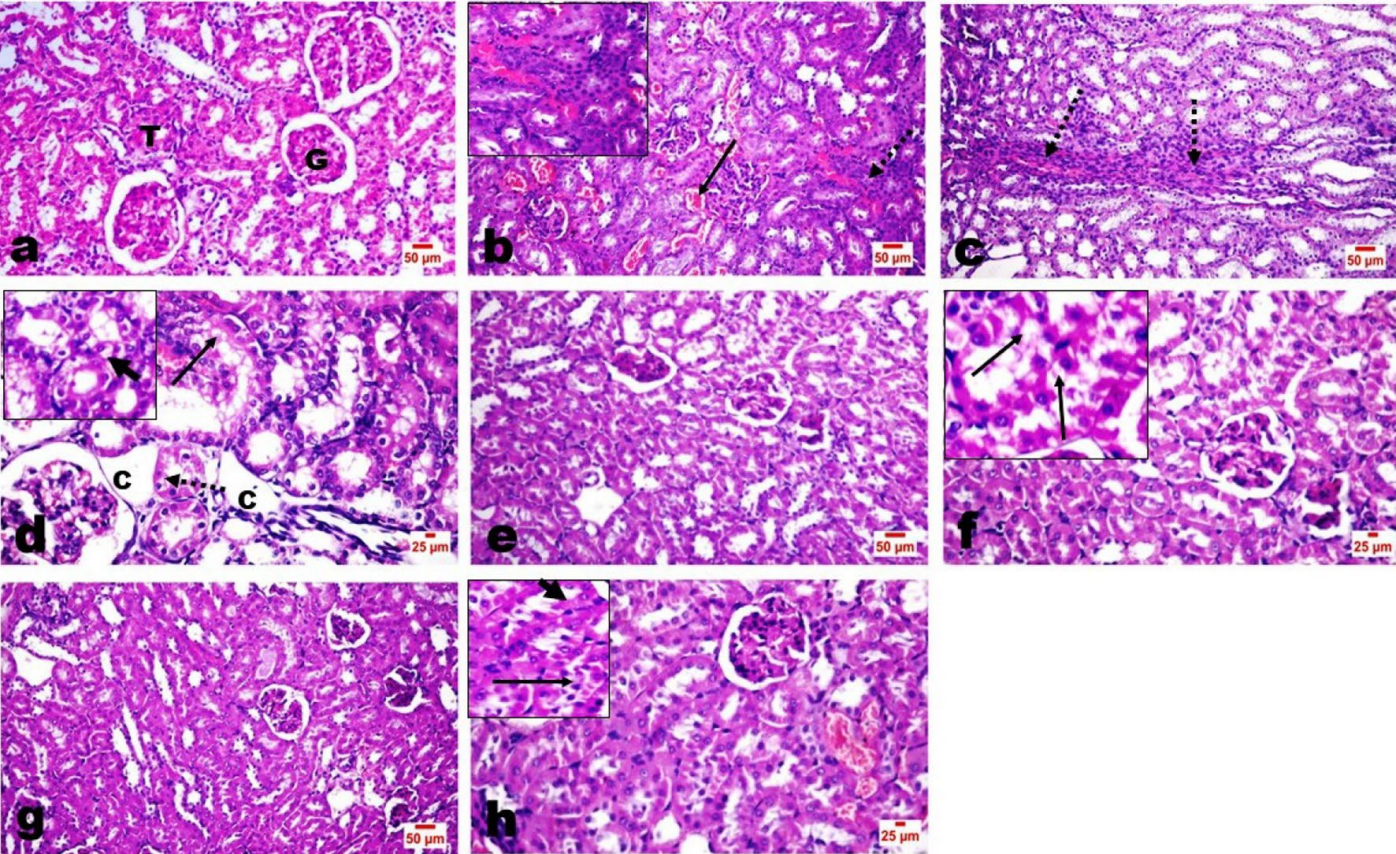
Effect of EPO on renal histopathology findings of TAA-intoxicated rats.

Kidneys of control rats had the normal histological structure of renal tubules, glomeruli (G), and interstitial tissue (T) (Fig. 6a). At the same time, the kidneys of TAA-administrated rats revealed mild to moderate corrugation of their capsule. The interstitial blood vessels were markedly congested (arrow) (Fig. 6b), with a moderate degree of fibroblastic proliferation among both cortical and medullary tubules (dotted arrow) (Fig. 6c). The renal tubular epithelial linings revealed degenerative changes, mostly vacuolar degeneration, apoptosis, nuclear pyknosis, and scattered single necrotic cells. Few tubules were cystically dilated with attenuated epithelium (Fig. 6d). Some tubules showed desquamation of some of their lining epithelial cells with attempts at granular cast formation.

Regarding the kidneys of EPO co-administered groups with TAA, the examination revealed against the action of TAA. No obvious fibroplasia could be detected in both groups. Mild to moderate degenerative changes of the renal tubular epithelium with some desquamated cells were the conspicuous findings with scattered apoptotic cells (Fig. 6e–h).

H&E-stained kidney micrographs. (a) kidney of a control rat showing normal glomeruli (G), tubules (T), and interstitial tissue. (b–d) kidney of TAA administrated rat showing congested interstitial blood vessels (arrow) and fibroblastic proliferation among both cortical (insert) and medullary (dotted arrow) tubules, (d) tubular epithelial vacuolar degeneration (thick arrow), apoptosis (arrow), and scattered necrosis (dotted arrow), and some cystically dilate (C) tubules. Low (d and e) and high (f and g) dose EPO-treated groups showed the absence of fibroplasia with mild degenerative changes (insert in f) of the tubular lining epithelial cells with some desquamated cells (insert in g).

### Immune histochemical findings

**Figure 7:**
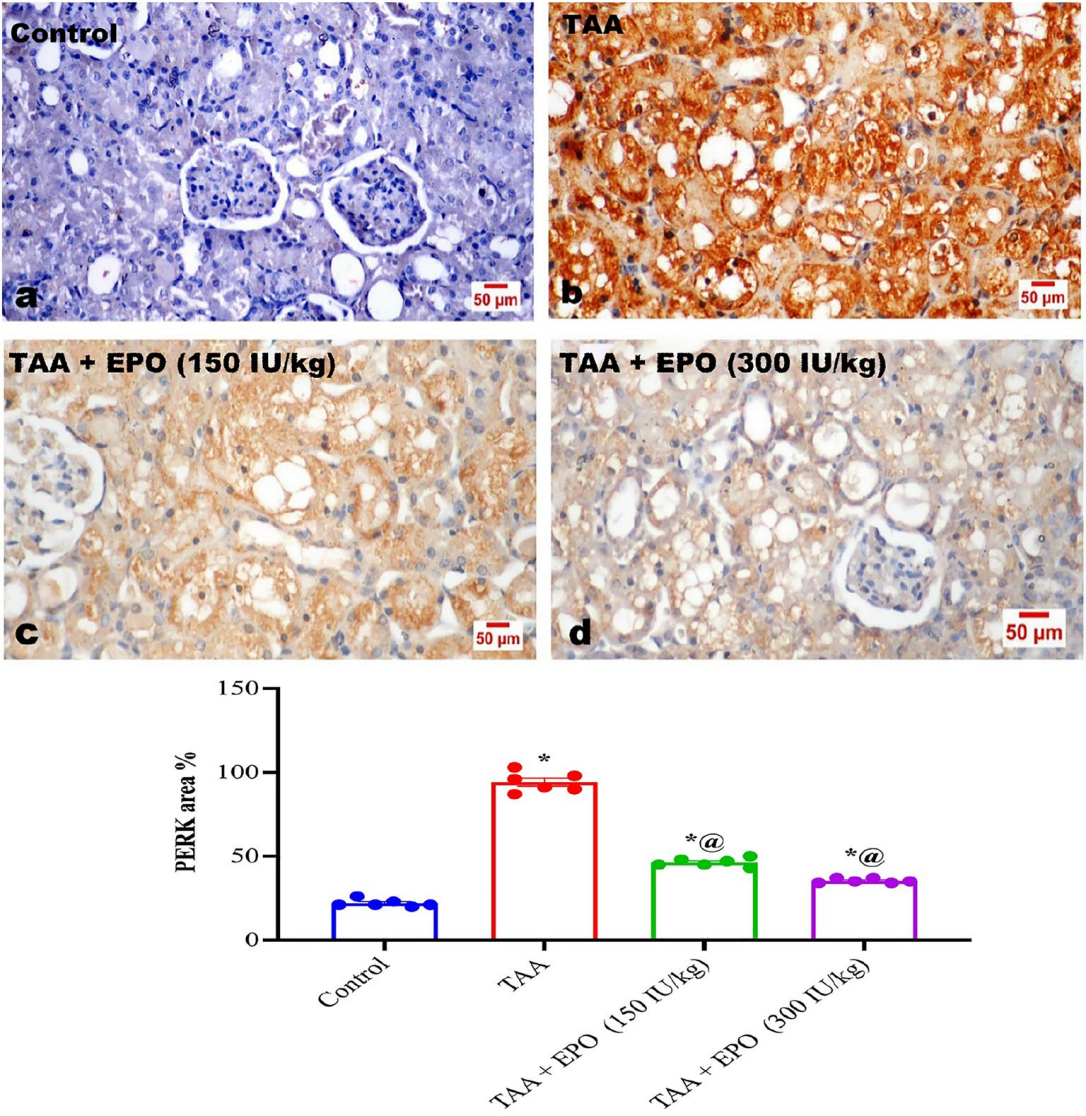
Effect of EPO on PERK expression in kidney tissues of TAA-intoxicated rats.

Immunohistochemical staining for detecting PERK expression in kidney tissues of various experimental groups revealed negative expression in the kidneys of control rats (Fig. 7a). Marked expression of PERK was noticed in the tubular epithelial linings in the kidneys of TAA-administrated rats (Fig. 7b) compared to control and various treated groups. EPO co-administration with TAA could deviously decrease the expression of PERK among the tubular epithelial linings in a dose-related pattern (Fig. 7c and d), as presented by the quantitative analysis of the positive expression by image analysis software (Fig. 7e).

Immunohistochemical micrographs for PERK expression in kidney tissues. (a) negative expression in the kidney of the control rat. (b) marked expression of PERK in the cytoplasm of tubular lining epithelium. (c) low and (d) high dose EPO treated groups showing decreased expression of PERK among the tubular epithelial linings in a dose-related pattern. (e) The quantitative analysis of the positive brown color of PERK expression in kidney tissue of all experimental groups by the image analysis software. Data are presented as mean ± SE. * vs. normal control group, @ vs. TAA group, # vs. EPO (150 IU/kg) at *p* < 0.05. EPO: Erythropoietin; TAA: thioacetamide.

## Discussion

The present study shows that EPO treatment reversed the toxic effect of TAA on the renal tissue and normalized renal functions, as demonstrated by serum BUN and creatinine levels. The neutralization of oxidative burden was the main finding and was recognized by normalizing MDA levels and increasing GSH content. Further, AMPK and JAK2/STAT5 signaling pathways were increased in the TAA group and were downregulated to normal levels after two weeks of treatment in EPO-treated groups. In the same context, TAA induced ER stress in kidney tissue and was reversed by EPO treatment, as shown by the ER stress marker PERK. There is evidence that the dynamics of AMPK and JAK2/STAT5 signaling are involved in EPO physiological effects. However, the effects of these signaling pathways and the link to EPO in the pathophysiology of renal tissue injury and loss of renal function have never been elucidated in vivo through the context of xenobiotic toxicity.

Regarding the results of EPO treatment on liver toxicity by thioacetamide, Ahmad et al.^34^ found that TAA-induced acute liver damage generates HIF-1α dependent rescue mechanisms with the translocation of EPO from the cytoplasmic to the nuclear compartment. Nuclear expression of EPO could explain its protective role during inflammatory and not only in hypoxic stress conditions within the liver. Moreover, large controlled and randomized clinical trials are still awaited to confirm the positive impact of experimental data and to solve problems we are currently facing on the use of drugs to treat AKI in common practice, e.g., adjusting doses in renal impairment, investigating combination therapies or testing the drugs in patients with other comorbid factors.

The current work shows that rats intoxicated with TAA have a marked impairment in renal function, which is supported by an increase in blood creatinine and BUN levels, as well as histological abnormalities. Previous researches suggested that using TAA could affect kidney function measures^6,35^. However, the administration of EPO in rats conserved renal function markers and prevented disruptions of its histological structure. Our findings are in accordance with^36^. Screening tests for renal function include serum urea and creatinine. Due to their largely glomerular filtration management and lack of renal control or adaptation in the face of diminishing renal function, they are excellent surrogates for GFR. Until more than half of renal function is lost, their readings stay within the normal range. However, within that range, a doubling of the readings (such as a jump from 8 to 16 mg/dl in BUN or from 0.6 to 1.2 mg/dl in sCr) may result in a 50% drop in GFR^37^. In the current study, creatinine and urea levels in the blood were dramatically elevated by 4.8 and 2.6-fold compared to the normal group after TAA was administered, as seen in Fig. 1.

Higher MDA levels and GSH consumption support the findings of the current study on TAA-induced oxidative stress. Prior studies similarly confirmed these findings^35,38–40^. As was previously indicated, TAA is converted in vivo to the free radical derivatives TAA sulfoxide and TAA-S, S-dioxide. This causes enhanced lipid peroxidation, which leads to the generation of reactive oxygen species (ROS), which causes damage to the kidney^7^. TAA can cause a significant amount of ROS to be produced, which can hinder the antioxidant defense system. Glutathione (GSH) and other thiols are components of the intracellular antioxidant system and may not be adequate to repair this damage^9^. EPO administration was found to decline MDA levels and enhance GSH content, improving the antioxidant status in kidney tissues. In addition, cisplatin-induced renal damage in rats was prevented by EPO administration via increasing the antioxidant status in kidney tissues^41^. The ability of EPO to prevent kidney damage may be due to its ability to reduce oxidative stress^42^.

**Figure 8:**
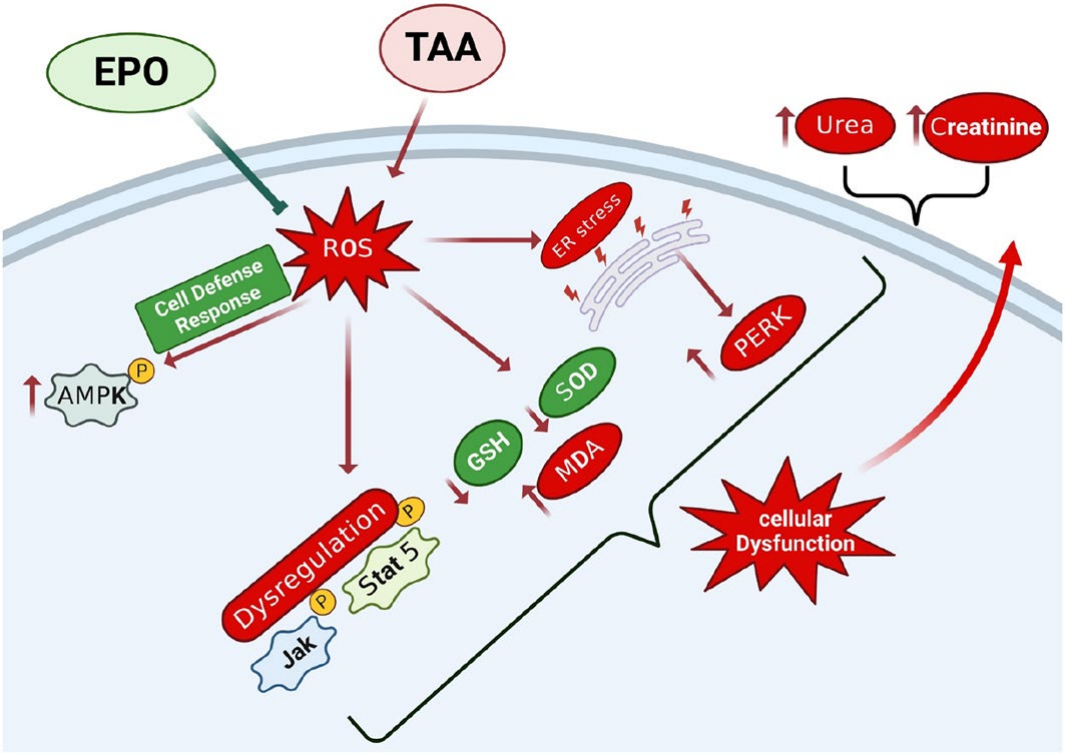
Protective effect of EPO against TAA-induced renal injury. EPO: Erythropoietin; TAA: thioacetamide; ROS: reactive oxygen species.

We postulated that the protective benefits of EPO against TAA-induced kidney damage could be linked to its ability to regulate the JAK/STAT system. To validate our results, JAK2 and STAT5 and their phosphorylated forms were measured in the kidneys of TAA- and EPO-treated rats. Recently, numerous studies have implicated the role of the JAK2/STAT5 signaling pathway in the progression of cellular apoptosis, inflammatory responses, and oxidative stress reactions^15^. Furthermore, the JAK2/STAT5 signaling system regulates cytokine activation, which regulates various cellular and immunological activities^43^. It has been shown in prior research that inhibiting the JAK2/STAT5 signaling pathway decreases transforming growth factor beta 1 (TGFβ1) production^44,45^. In the present study, TAA rats demonstrated a significant increase in renal content of JAK2/p-JAK2 and STAT5/p-STAT5, as shown in Fig. 8. In addition, the upregulation of the JAK2/STAT5 pathway via TAA was previously studied^46^.

Meanwhile, EPO administration could down-regulate the JAK2/STAT5 signaling pathway compared with TAA rats. Accordingly, we propose that the downregulation of JAK2/STAT5 signaling is due to the time and dose of EPO. Additionally, we report that EPO does not augment JAK2/STAT5 in the absence of oxidants burden, as demonstrated after the remission of the ROS burden in EPO-treated groups. Notably, EPO signaling and its link to JAK/STAT5 signaling has been studied most thoroughly in erythroid progenitor cells of the bone marrow. The binding of EPO to its receptor causes autophosphorylation of JAK2 during erythropoiesis^47^. STAT5 is recruited, phosphorylated, dimerized, and translocated to the nucleus to initiate transcription of the Bcl2 family of genes that inhibit erythroid progenitor apoptosis^48^. Although the JAK2/STAT5 signaling axis is considered a major EPO signaling pathway for erythropoiesis, recent research suggests that it is more crucial for erythropoiesis under stress conditions^49^.

New data suggests that JAK2-STAT5 crosstalk may involve the G-protein coupled receptor (GPCR) and phosphoinositide 3-kinases (PI3K) signaling pathways. Interactions with JAK2-STAT5 signaling pathways are quite intricate^50^. Besides, it was reported that the activation of the Pi3K/AKT pathway slows down tubular healing and increases renal fibrosis in IR injury-induced acute kidney injury. On the contrary, the treatment with EPO in the same model is associated with the prevention of programmed cellular death^51^. Our outcomes adhere to the aforementioned data in which the TAA-enhanced renal injury was associated with the activation of JAK2/PI3K/STAT5, another indirect pathway of STAT5 activation. Interestingly, in the current study, EPO protected the renal damage by halting the JAK2/PI3K/STAT5. This result was in agreement with Yu et al. in which EPO protected EPO the epithelial cells from undergoing programmed cell death and autophagy during experimental newborn necrotizing enterocolitis^52^.

TAA toxicity leads to augmented oxidative burden and loss of oxidative/ antioxidants balance. We report that TAA toxicity stimulates AMPK expression and AMPK phosphorylation. AMPK activation is thought to be directly involved in neutralizing this toxicity. Downstream to AMPK, multiple effectors act as cellular defenses against oxidative stress^53^. Our study showed that two weeks of EPO administration down-regulated the induction of AMPK/p-AMPK signaling by TAA. This observation could be interpreted by increasing the intracellular antioxidant capacity by the upregulation of antioxidant enzymes directly by EPO. Consequently, decreasing ROS levels and relief of AMPK/p-AMPK from ROS-induced upregulation. Importantly, this interpretation comes from the evidence that EPO acts as an antioxidant either directly through the scavenging action done by using its sugar moiety or indirectly through the activation of antioxidant effectors enzymes such as GSH, SOD, and catalase^35,54–58^.

Several renal illnesses have been linked to endoplasmic reticulum (ER) stress, and this link has been well-validated^59^. Xiao et al. stated that “Cells respond to ER stress by activating the unfolded protein response (UPR), and glucose-regulated protein of 78 kDa (GRP78) serves as a central regulator of three main UPR sensors, namely, activating transcription factor (ATF)6, inositol-requiring enzyme (IRE)-1α, and protein kinase RNA-like ER kinase (PERK), that initiate the UPR signaling pathway under ER stress”^60^. Proteinuric kidney disorders, such as diabetic nephropathy and human immunodeficiency virus-related nephropathy, were correlated with increased expression of these ER stress indicators in the tubulointerstitial compartment of kidney cells^61,62^. Immunohistochemical staining findings showed that EPO downregulated PERK expression, which was increased in TAA rats. Previous research has shown that EPO can reduce intracellular ER stress, protecting rats against cardiac failure and nephrotoxicity^63,64^.

The Protein Kinase R-like ER Kinase (PERK) is an important protein located in the ER that regulates signal transduction during ER stress. Together with activating transcription factor 6 (ATF6) and inositol-requiring enzyme 1 alpha (IRE1α), PERK is recognized as a key effector and marker of ER stress^65^. ER stress is closely associated with the overwhelming oxidative burden^66^. Importantly, activation of AMP-activated protein kinase inhibits ER stress in the process of development of renal injury. In the present study, we investigated PERK expression upon TAA administration and in response to EPO treatment, aiming to have an indicator of ER stress. We showed that EPO inhibited ER stress induced by TAA. This effect may be downstream of AMPK activation dynamics^67^.

The generation of red blood cells is controlled by two different kinds of erythropoietin receptors (EpoR), EPOR and EPOR2^68^. EPOR isoforms may explain the nonhematopoietic action of EPO in distinct tissues. Previous investigations have shown many EPOR isoforms, including canonical homodimer (EPOR2); EPOR is physically interacting with a beta common receptor, a receptor for cytokines (EPOR/βcR); the EPOR/βcR heterodimer has been reported to protect tissues, unlike EPOR2, which is involved in erythropoiesis^69,70^. In βcR-knockout mice, cardiomyocytes and spinal cord damage models supported the idea that EPOR/βcR is a tissue-protective heteroreceptor^71^.

Nevertheless, prolonged exposure to EPO in some patients may suffer from a rise in blood pressure. Higher dosages or prolonged usage of EPO are more likely to cause this side effect. Careful monitoring of blood pressure and titration of EPO dose as appropriate allows for effective management of hypertension^72^. Furthermore, thromboembolic events, such as deep vein thrombosis (DVT) and pulmonary embolism, have been linked to EPO medication. Patients with preexisting cardiovascular disease or a family history of thrombotic events are at a much higher risk for this^73^.

## Conclusions

The present study documents that AMPK and JAK2/STAT5 signaling pathways in the renal tissue are augmented in TAA toxicity. The phenotype we show that two weeks of EPO treatment neutralized oxidative burden and downregulated AMPK and JAK2/STAT5 and the JAK2/PI3K/STAT5 defensive signaling and switching the cellular environment from anti-ROS defense to a house-keeping mode of activity, those molecular events are describing EPO effect in inhibition of the renal injury and restoration of kidney function. Our study suggests that EPO is recommended to be tested in trials for the purpose of renal protection against clinical situations of biological oxidative insulation of kidney tissue.

## Limitations of the current study

More studies are warranted to explore the association of EPOR/βcR in the reno-protective effect of EPO. Additionally, this work does not give a thorough molecular knowledge of how EPO exerts its protective benefits; nevertheless, it does highlight the possible participation of multiple signaling pathways (JAK2/STAT5, AMPK, PI3K, PERK) and their regulation by EPO. More research is required to understand the underlying molecular processes.

## Data Availability

Data will be available upon request from Marawan A Elbaset.
